# Fibrin-binding, peptide amphiphile micelles for targeting glioblastoma^☆^

**DOI:** 10.1016/j.biomaterials.2013.10.064

**Published:** 2013-11-07

**Authors:** Eun Ji Chung, Yu Cheng, Ramin Morshed, Kathryn Nord, Yu Han, Michelle L. Wegscheid, Brenda Auffinger, Derek A. Wainwright, Maciej S. Lesniak, Matthew V. Tirrell

**Affiliations:** a Institute for Molecular Engineering, The University of Chicago, 5747 S. Ellis Ave. Jones 222, Chicago, IL 60637, USA; b The Brain Tumor Center, The University of Chicago, 5841 S. Maryland Ave. MC3026, Chicago, IL 60637, USA

**Keywords:** Self-assembly, Micelle, Brain, Diagnostic, Glioblastoma, Targeting

## Abstract

Glioblastoma-targeted drug delivery systems facilitate efficient delivery of chemotherapeutic agents to malignant gliomas, while minimizing systemic toxicity and side effects. Taking advantage of the fibrin deposition that is characteristic of tumors, we constructed spherical, Cy7-labeled, targeting micelles to glioblastoma through the addition of the fibrin-binding pentapeptide, cysteine–arginine–glutamic acid–lysine–alanine, or CREKA. Conjugation of the CREKA peptide to Cy7-micelles increased the average particle size and zeta potential. Upon intravenous administration to GL261 glioma bearing mice, Cy7-micelles passively accumulated at the brain tumor site via the enhanced permeability and retention (EPR) effect, and Cy7-CREKA-micelles displayed enhanced tumor homing via active targeting as early as 1 h after administration, as confirmed via *in vivo* and *ex vivo* imaging and immunohistochemistry. Biodistribution of micelles showed an accumulation within the liver and kidneys, leading to micelle elimination via renal clearance and the reticuloendothelial system (RES). Histological evaluation showed no signs of cytotoxicity or tissue damage, confirming the safety and utility of this nanoparticle system for delivery to glioblastoma. Our findings offer strong evidence for the glioblastoma-targeting potential of CREKA-micelles and provide the foundation for CREKA-mediated, targeted therapy of glioma.

## 1. Introduction

Gliomas are primary brain tumors that are derived from astrocytes, oligodendrocytes, or ependymal cells. Glioblastoma multiforme (GBM), a grade IV astrocytoma, accounts for 70% of all malignant gliomas and carries a 5-year survival rate of less than 5% with a 14.6-month median survival time even with the treatment of aggressive radiotherapy and concomitant or adjuvant temozolomide [1,2]. Treatment of glioblastoma represents one of the most formidable challenges in oncology due to the inability to completely surgically resect the tumor owing to a diffuse invasion of the surrounding normal brain tissue. The amount of tumor removed is also limited by a proximity to critical regions for brain function, unavoidably resulting in tumor recurrence [3]. Consequently, post-surgical chemotherapy regimens are considered to be essential for the treatment of gliomas, but therapeutic advancement for GBM has remained disappointing due to poor penetration of chemotherapy agents successfully crossing the blood–brain-barrier (BBB) [4]. Although this interface acts to protect the brain from exposure to harmful substances circulating in the bloodstream under normal physiological conditions, the BBB simultaneously limits penetration of drugs to cancerous tissue, even when partially compromised [5,6].

Recent developments in nanoparticle technology have yielded promising results in the delivery of therapeutic agents across the BBB [7–9]. Micelles are of particular interest due to their small size, biocompatibility, and hydrophobic core, which is especially advantageous for loading poorly soluble drugs [10]. When properly designed, micelles can prolong drug bioavailability in circulation and deliver drugs in the optimum dosage range, which results in improved therapeutic efficacy and reduced toxic side effects [11]. Importantly, micelles have been shown to chaperone chemotherapy agents across the BBB as active targeting drug delivery systems in addition to passively targeting tumor tissue via the enhanced permeability and retention (EPR) effect [12,13]. Through surface-modifications with ligands such as transferrin, epidermal growth factor, folate, and α2-glycoprotein, micelles can bind to receptors that are selectively expressed on cancer cells or the tumor vasculature [10,13–15].

**Fig. 1:**
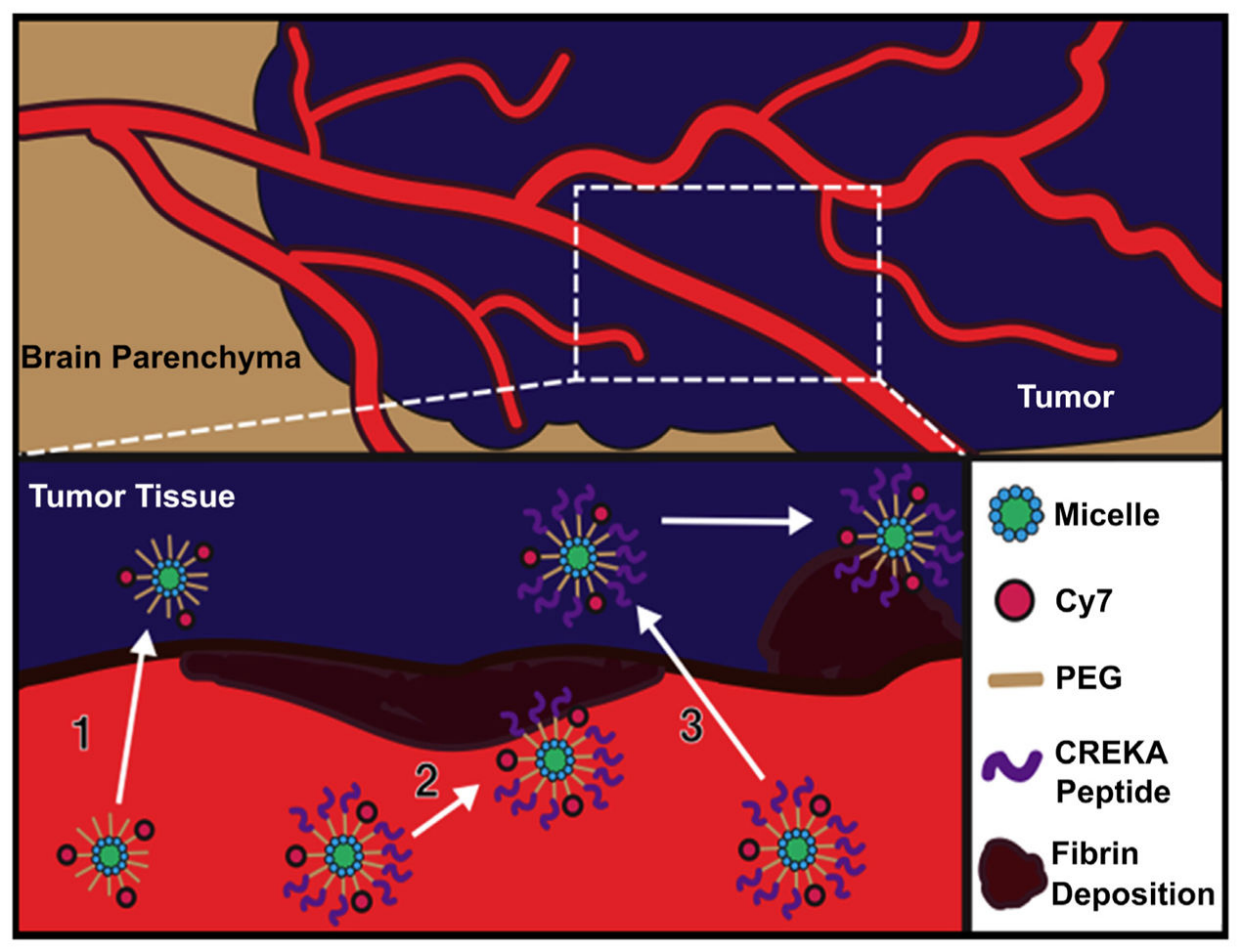
Schematic of Cy7-micelle and Cy7-CREKA-micelle targeting glioma via the EPR effect and active binding to fibrin.

Previous reports demonstrate GBM is accompanied by leaky, hemorrhagic vasculature, which contributes to thrombosis and fibrin deposition [16]. The presence of fibrin and proteins that become cross-linked to fibrin in tumors, but not in normal tissues, are thought to be a result of the leakiness of tumor vessels, which allows plasma proteins to enter from the blood into tumor tissue where fibrinogen is converted to fibrin via tissue pro-coagulant factors [17]. Since fibrin deposition within and around the tumor area is characteristic to primary and metastatic brain tumors, we examined the targeting capabilities of micelle nanoparticles functionalized with the clot-binding peptide, cysteine–arginine–glutamic acid–lysine–alanine (CREKA) [18–20]. In our earlier studies, fluorescently-labeled micelles functionalized with the CREKA pentapeptide were reported to home to sites of fibrin found on thrombotic, atherosclerotic plaques [21]. Furthermore, CREKA has also been shown to target breast and prostate cancer by binding to fibrin and fibrin-associated clotted plasma proteins within the tumor vasculature [18,20]. For the first time, the potential of CREKA-micelles as a targeting nanoparticle system for gliomas via active binding to fibrin within the tumor blood vessels was investigated in this study. To this end, peptide amphiphiles consisting of the CREKA peptide were used to self-assemble targeting micelles. We intravenously injected Cy7 labeled micelles with or without the CREKA peptide to mice bearing intracranial glioma tumors and hypothesized that the micelles would be capable of targeting brain tumor tissue through the EPR effect and that the CREKA modification could further enhance tumor homing of the micelles by binding to intravascular clots as well as interstitial areas of fibrin deposition (Fig. 1). In addition to targeting, the clearance properties of these micelles were evaluated for up to 7 days.

## 2. Materials and methods

### 2.1. CREKA micelle synthesis

The [Cys–Arg–Glu–Lys–Ala] peptide was synthesized using standard Fmoc-mediated solid phase peptide synthesis methods on rink Amide resin (Anaspec, Fremont, CA, USA) using an automated PS3 Benchtop Peptide Synthesizer (Protein Technologies, Tucson, AZ, USA). Peptides were cleaved and deprotected with 94:2.5:2.5:1 by volume trifluoroacetic acid: 1,2-ethanedithiol:H_2_O:triisopropylsilane and were precipitated and washed several times with cold diethyl ether, dissolved in water, lyophilized, and stored as lyophilized powders at 20 °C. Crude, peptide mixtures were purified by reverse-phase HPLC (Prominence, Shimadzu, Columbia, MD, USA) on a C8 column (Waters, Milford, MA) at 55 °C using 0.1% trifluoroacetic acid in acetonitrile/water mixtures and characterized by MALDI-TOF/TOF mass spectral analysis (Biflex III, Bruker, Billerica, MA, USA). Cysteine-containing peptides were conjugated via a thioether linkage to DSPE-PEG(2000)-maleimide (Avanti Polar Lipids, Alabaster, AL, USA) by adding a 10% molar excess of the lipid to peptide in water. After reaction at RT for 24 h, the resulting product was purified and characterized as described above. Cy7 was conjugated via a peptide bond to DSPE-PEG(2000)-amine (Avanti Polar Lipids, Alabaster, AL, USA) by adding an equal molar equivalent of Cy7 mono-N-hydroxysuccinimide ester (GE Healthcare Life Sciences, Pittsburgh, PA, USA) to the lipid dissolved in 10 mM aqueous sodium carbonate buffer (pH 8.5). After reaction at RT for 24 h, the mixture was also purified on a C4 column and characterized as described above.

CREKA micelles were assembled by dissolving the Cy7 and peptide containing DSPE-PEG(2000) amphiphiles in methanol (10:90 molar ratio), mixing the components, and evaporating the mixed solution under nitrogen. The resulting film was dried under vacuum O/N, and then hydrated at 80 °C for 30 min in water or PBS and allowed to cool to RT. Non-targeting micelles were assembled by combing DSPE-PEG(2000)-Cy7 and DSPE-PEG(2000)-maleimide.

### 2.2. Micelle characterization

#### 2.2.1. Critical micelle concentration determination via 1,6-diphenyl-1,3,5-hexatriene (DPH) fluorescence

The critical micelle concentration (CMC) was determined using DPH fluorescence [22]. This method relies on an increase in DPH fluorescence as it partitions from aqueous solution into the hydrophobic micelle core. Solutions of the CREKA peptide amphiphile (PA) ranged in concentrations from 316 to 0.01 μM. DPH was dissolved in tetrahydrofuran (THF) and a small amount of this solution was added to each PA solution to achieve 1 μM DPH with a residual THF volume percentage of approximately 0.1. Solutions containing PA and DPH were allowed to equilibrate for 1 h prior to measurement using a Tecan Infinite 200 plate reader (Mannedorf, Switzerland). DPH was excited at 350 nm and fluorescence emission was measured at 428 nm.

#### 2.2.2. Dynamic light scattering (DLS)

Stock solutions of 100 μM DSPE-PEG(2000)-Cy7 and DSPE-PEG(2000)-CREKA in PBS were used to confirm the presence of small spheroidal micelles and DLS measurements were determined at 90 ° and 637 nm using a Brookhaven Instruments (Holtzville, NY, USA) system consisting of a BI-200SM goniometer and a BI-9000AT autocorrelator.

#### 2.2.3. Transmission electron microscopy (TEM)

Negative staining samples for TEM were prepared by placing the CREKA PA solution on 400 mesh lacey carbon grids (Ted Pella, Redding, CA, USA) for 2 min. Excess liquid was wicked away with filter paper and the grid was washed with Milli-Q water before placing 1 wt.% phosphotungstic acid solution for 2 min and washing with Milli-Q water. Dried samples were imaged on a JEOL 1230 TEM, immediately (JEOL, Ltd., Tokyo, Japan).

#### 2.2.4. Zeta potential

Zeta potential of 100 μM DSPE-PEG(2000)-Cy7:DSPE-PEG(2000)-CREKA and DSPE-PEG(2000)-Cy7:DSPE-PEG(2000)-maleimide (10:90 molar ratio) dissolved in water were measured (Zetasizer Nano ZS, Malvern, Worcestershire, United Kingdom, *N* ≥ 3).

### 2.3. Animal experiments

To assess the targeting abilities of Cy7- and Cy7-CREKA-micelle nanoparticles, 8–10 weeks old male C57BL/6 mice (Cat# 000664; Jackson Laboratories, Bar Harbor, ME, USA) were intracranially injected with 4 × 10^5^ GL261 murine glioma cells suspended in 5 μl PBS into the right hemisphere of the brain. Tumors were allowed to grow for 1 week before administration of 100 μl of 1 mM Cy7-CREKA-micelles, Cy7-micelles, or PBS via tail vein injection. The fur was naired and removed, before obtaining *in vivo* images at 1, 2, and 3 h post-injection using a Xenogen IVIS 200 imaging system (Caliper Life Sciences, Hopkinton, MA). Mice were then euthanized via CO_2_ overdose and organs (e.g. brain, heart, lungs, liver, kidneys, spleen, large intestines, and bladder) were excised at 3 h, 24 h, and 7 days post-injection and imaged. Quantification and comparisons of the fluorescence signal were achieved via Living Image software (PerkinElmer, Downers Grove, IL, USA, *N* ≥ 3). In order to confirm that the micelles could target brain tumors and that the act of injecting did not allow for accumulation of micelles at the injury site, mice were intracranially injected with 5 μl PBS to establish the brain injury model, and Cy7- and Cy7-CREKA-micelles were administered and imaged after 24 h post-injection. All animal procedures followed NIH guidelines for the care and use of laboratory animals and were approved by the University of Chicago’s Institutional Animal Care and Use Committee (Chicago, IL, USA).

### 2.4. Immunohistochemistry and histology

Following optical imaging, the brain, liver, and kidney were immediately frozen and embedded in O.C.T. Tissue Tek (Tissue Tek, Sakura Finetek, Torrance, CA, USA) before 10 μm samples were sectioned (Microm HM 525, Fisher Scientific, Pittsburg, PA, USA). Tissue sections were stained with Hematoxylin & Eosin (H & E), scanned (CRi Panoramic, PerkinElmer, Downers Grove, IL, USA), and analyzed with Pannoramic Viewer 1.14.53.0 software (3DHISTECH, Budapest, Hungary). For immunohistochemistry, tissue sections were fixed with 4% paraformaldehyde, followed by incubation with Image-iT FX signal enhancer (Invitrogen, Carlsbad, CA, USA) for 30 min at RT. Sections were washed with PBS and incubated overnight at 4 °C with an antibody against fibrinogen (Santa Cruz Biotech, Santa Cruz, CA, USA, 1:100). The next day, sections were incubated for 1 h at RT using goat, anti-rabbit IgG-FITC (Santa Cruz Biotech, Santa Cruz, CA, USA, 1:100), and mounted using Molecular Probes ProLong Gold anti-fade reagent with DAPI (Invitrogen, Carlsbad, CA, USA).

### 2.5. Statistical analysis

A Student’s *t*-test was used to compare means of pairs. Analysis of variance (ANOVA) with Newman–Keuls multiple comparison test post-hoc analysis was used to determine significant differences among three or more means. A *p*-value of 0.05 or less was considered to be significant.

## 3. Results

### 3.1. Characterizations of micelles

**Fig. 2:**
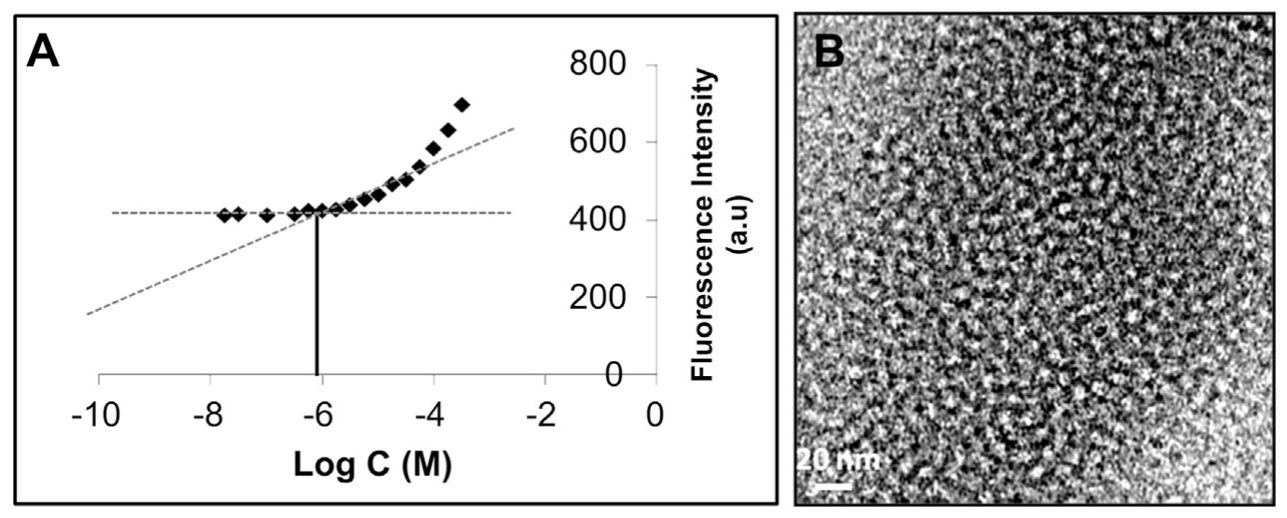
Characterization of CREKA-micelles. A) The CMC is approximately 1 μM and B) TEM images confirm the spherical shape of CREKA-micelles.

The fibrin-binding pentapeptide, CREKA, was conjugated to DSPE-PEG(2000)-maleimide via a thioether linkage to cysteine, and the CMC of resulting PAs was determined to be 9.3 × 10^−7^ μM (Fig. 2A). Fluorescently-labeled non-targeting (Cy7-micelle) and CREKA micelles (Cy7-CREKA-micelle) were constructed by mixing DSPE-PEG(2000)-Cy7 with either DSPE-PEG(2000)-CREKA or DSPE-PEG(2000)-maleimide in a 10:90 molar ratio, and the presence of spherical micelles with an average diameter of 7.6 ± 1.2 and 8.2 ± 1.2 nm was confirmed via TEM and DLS (Fig. 2B and Table 1). Zeta potentials of Cy7- and Cy7-CREKA-micelles were determined to be −27.4 ± 4.5 and −12.6 ± 1.6 mV, respectively (Table 1).

### 3.2. Micelle targeting to gliomas

**Fig. 3:**
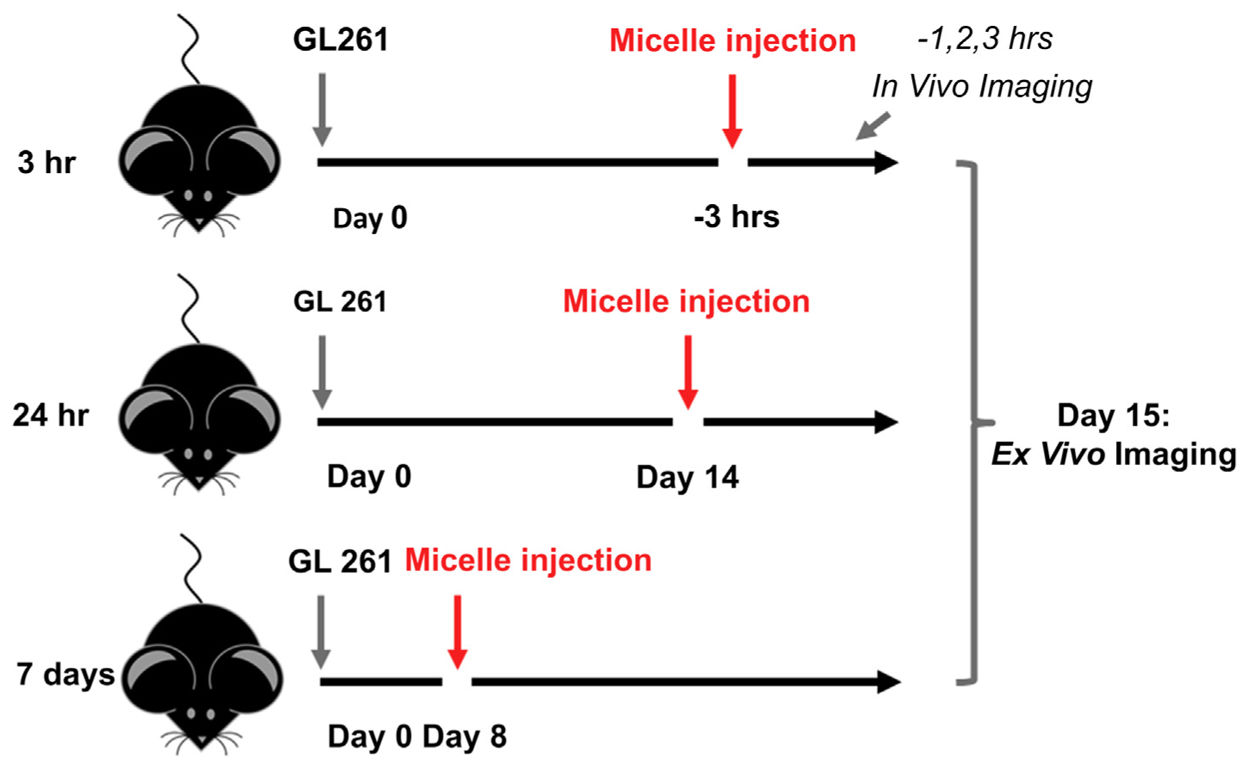
Design of animal studies.

**Fig. 4:**
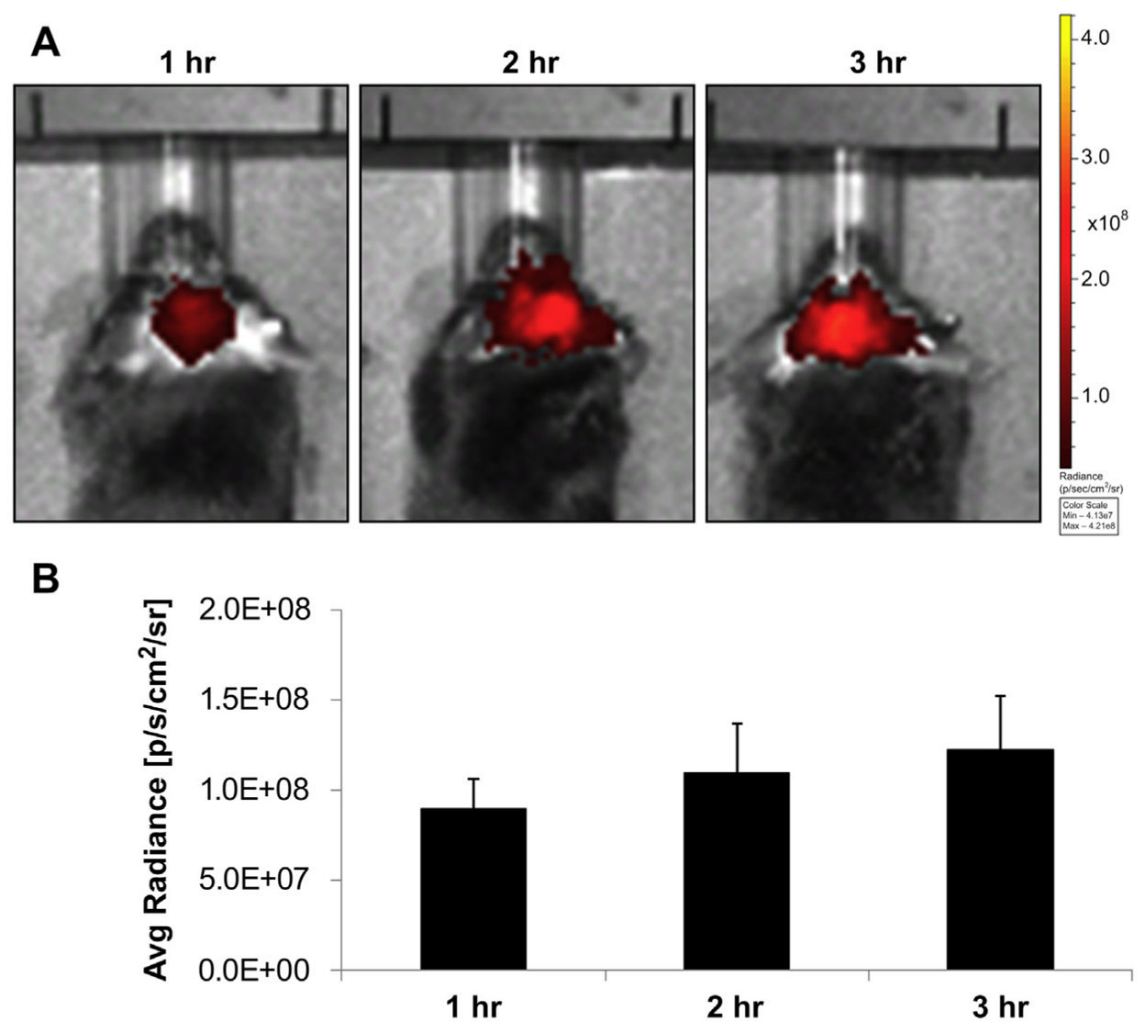
*In vivo* imaging of Cy7-CREKA-micelles. A) *In vivo* images show accumulation of Cy7-CREKA-micelles in the brain and B) quantification of the fluorescence signal confirms accumulation increases for up to 3 h.

**Fig. 5:**
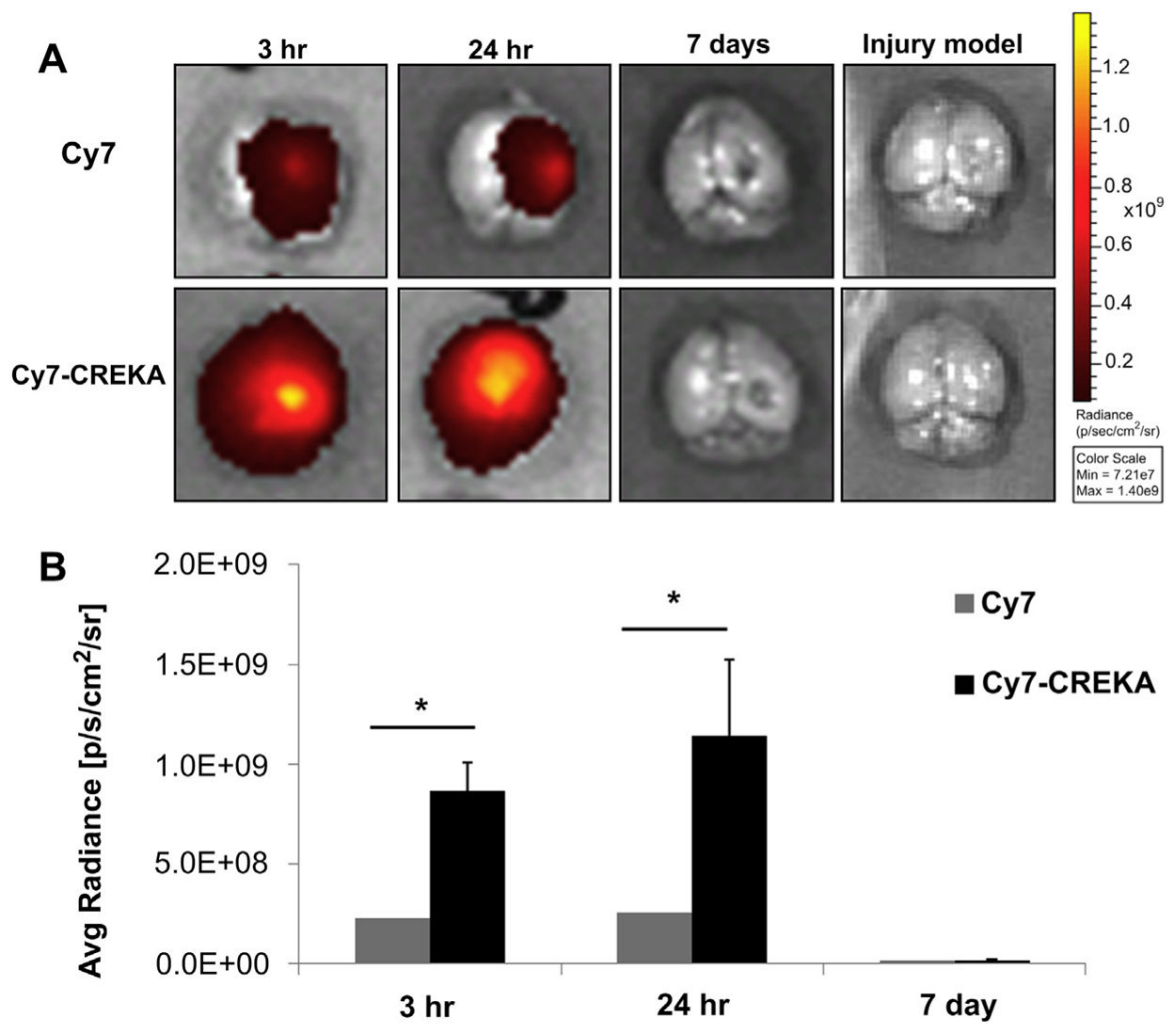
*Ex vivo* imaging of the brain. A) Cy7-micelles and Cy7-CREKA-micelles localize to brain tumor tissue and are cleared by 7 days. No accumulation is seen in the injury model after 24 h. B) Cy7-CREKA-micelles accumulate within tumor tissue to a greater extent than Cy7-micelles at 3 and 24 h post-injection. Asterisks denote statistical significance (*p* ≤ 0.05).

**Fig. 6:**
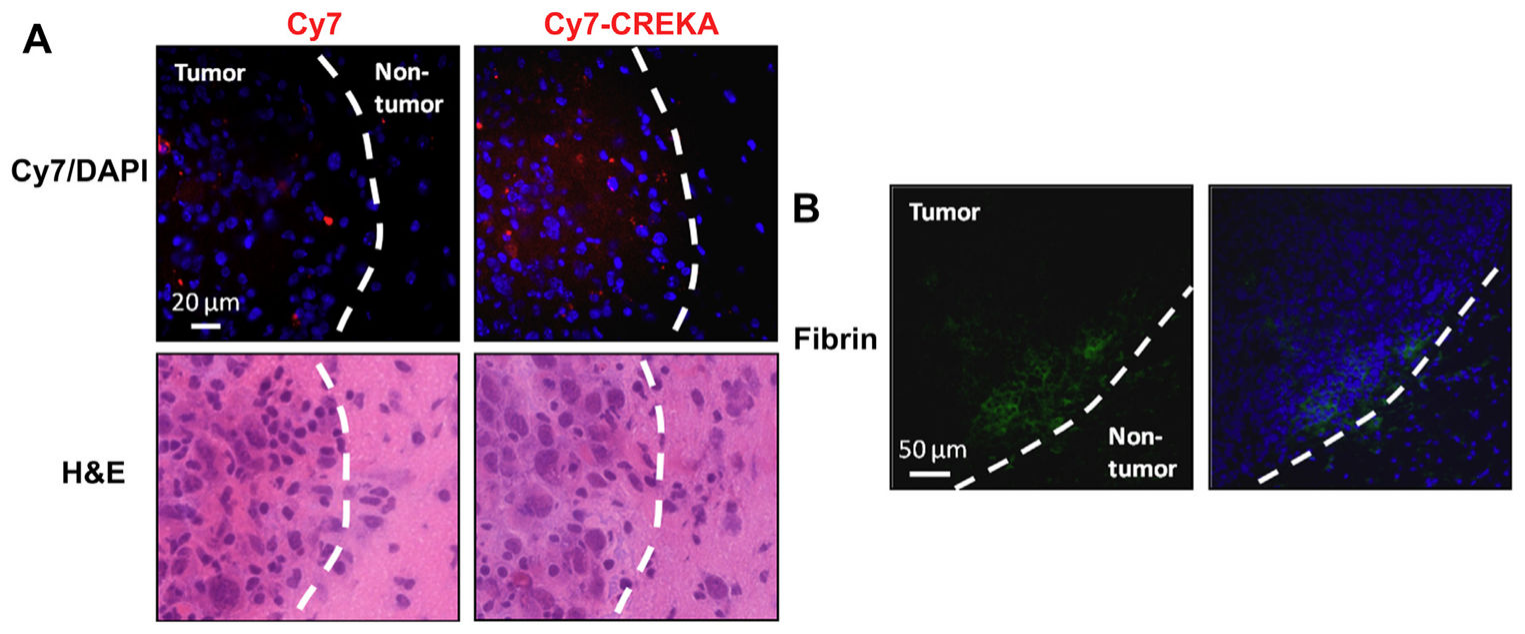
Histological assessment of micelle and fibrin distribution. A) Fluorescence images and H & E staining exhibit punctate patterns of the Cy7 signal within the tumor tissue in both Cy7-micelle and Cy7-CREKA-micelle treated mice. Additionally, dimmer areas of Cy7 were present in mice injected with Cy7-CREKA-micelles. No Cy7 was found in non-tumor tissues. B) Fibrin was present throughout the tumor region.

In order to assess passive targeting of Cy7-micelles via the EPR effect and enhanced accumulation of Cy7-CREKA-micelles, micelles were intravenously administered in an intracranial GL261 glioma mouse model and assessed via optical imaging (Fig. 3). After 1 h in blood circulation, the localization of Cy7-CREKA-micelles at the tumor site was visible via *in vivo* imaging and the accumulation increased with time (Fig. 4). When brains were excised and imaged, both Cy7-micelles and Cy7-CREKA-micelles were localized at the tumor site within 3 h post-injection and cleared by 7 days (Fig. 5A). In contrast, no fluorescence signal was found when micelles were administered in a brain injury model (Fig. 5A). Both Cy7- and Cy7-CREKA-micelles demonstrated increased accumulation from the 3 h–24 h time points, but Cy7-CREKA-micelles accumulated to a significantly greater extent than their non-targeting counterparts at each time point (3 h: 8.7 × 10^8^ vs. 2.3 × 10^8^ p/s/cm^2^/sr; 24 h: 1.6 × 10^9^ vs. 1.7 × 10^8^ p/s/cm^2^/sr; Fig. 5B). When samples were sectioned and evaluated histologically, both Cy7- and Cy7-CREKA-micelles showed bright, individual, punctate patterns of Cy7 fluorescence within the tumor region of the brain, and no Cy7 signal was found in the non-tumor tissue (Fig. 6A). Notably, Cy7-CREKA samples also showed a dimmer fluorescence signal present throughout the sample. Immunohistochemistry also confirmed a generous amount of fibrin to be present within the tumor tissue (Fig. 6B).

### 3.3. Biodistribution of micelles

**Fig. 7:**
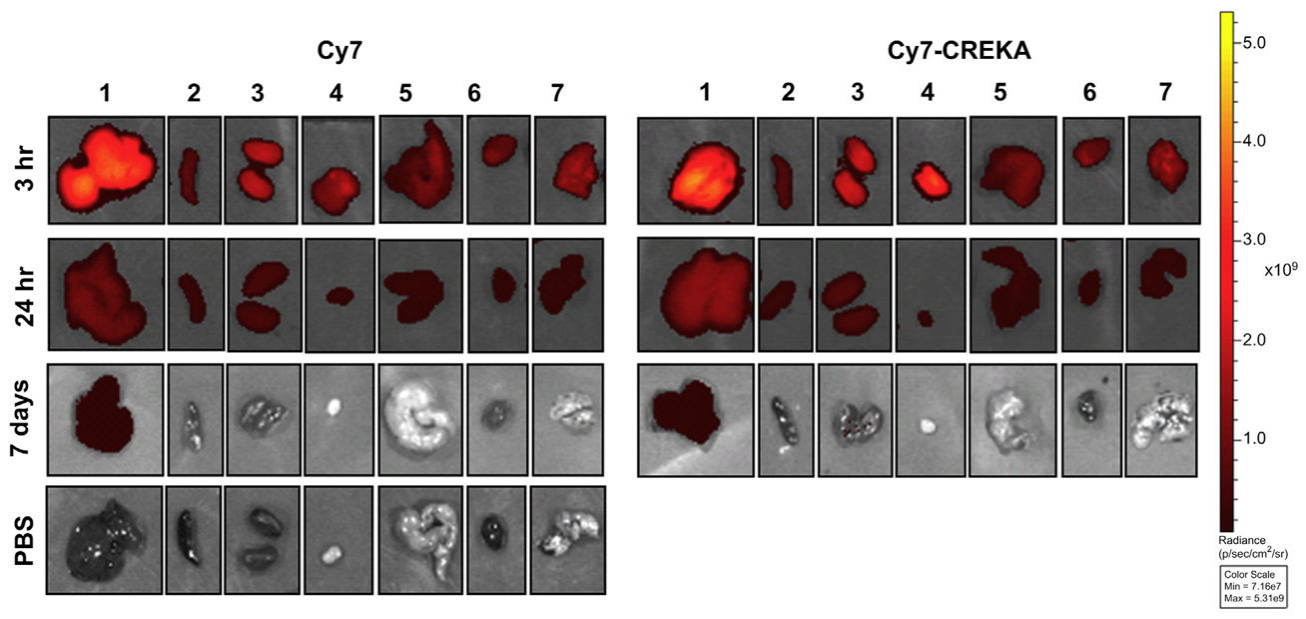
Biodistribution of micelles. Fluorescence was measured at 3 h, 24 h, and 7 day time points in (1) liver, (2) spleen, (3) kidneys, (4) bladder, (5) large intestines, (6) heart, and (7) lungs. Micelles localized primarily to the liver, kidneys, and bladder.

Fig. 7 shows the biodistribution of micelles throughout the liver, spleen, kidneys, bladder, large intestines, heart, and lung at 3 h, 24 h, and 7 days after injection using optical imaging (Fig. 7). At all time points, both Cy7- and Cy7-CREKA-micelles were found to be primarily localized in the liver. Micelles also showed greater accumulation in the kidney and bladder when compared to the rest of the organs. By 7 days, both types of micelles were mostly cleared from circulation and were found to reside only within the liver.

### 3.4. Liver and kidney assessment

**Fig. 8:**
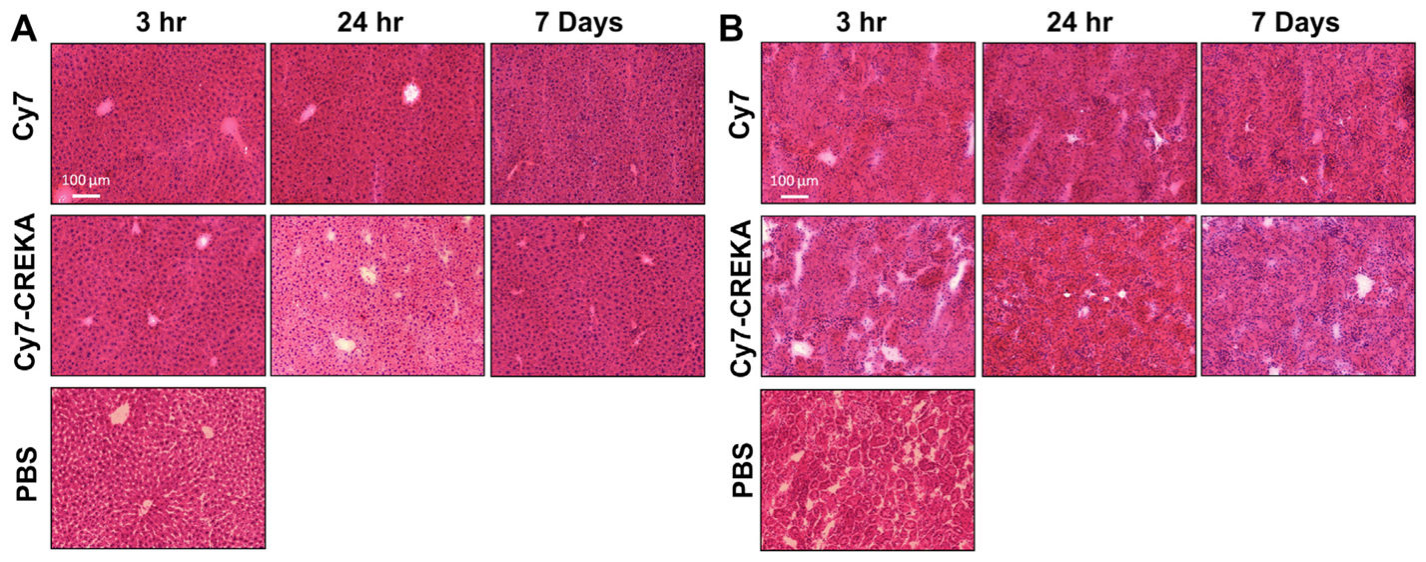
Representative H and E staining of the liver and kidney upon micelle administration. No morphological changes or tissue damage was demonstrated in A) livers and B) kidneys treated with micelles in comparison to the PBS-treated controls.

As the liver and kidney are vital organs for metabolism and excretion, tissues were stained with H & E, examined using light microscopy, and representative images from mice treated with PBS, Cy7-micelles, or Cy7-CREKA-micelle at all time points are presented. As seen in Fig. 8, no apparent tissue or cellular damage was observed in mice injected with either micelles and were comparable to images obtained from mice injected with PBS, and no pathological changes were observed.

## 4. Discussion

The treatment of glioblastoma is limited due to the aggressive nature of the tumor and the inability for complete surgical excision. As a result, GBM prognosis remains poor, with a 14.6 month median survival time despite interventions [1]. Chemotherapy against glioblastoma has not translated into meaningful improvements in patient outcome due to low permeability and poor penetration across the BBB [23,24]. Furthermore, systemically delivered chemotherapy poses challenges including high toxicity and numerous side effects [24]. In contrast, the rational design of targeted delivery vehicles has the potential to efficiently unload drugs to the tumor site, while sparing the surrounding healthy tissue and the function of vital organs.

In this study, fluorescently-labeled micelle nanoparticles functionalized with the pentapeptide, CREKA, were intravenously injected in a mouse model of glioma. Since CREKA has been shown to target other types of tumors *in vivo* by binding to fibrin intra-vascularly and fibrin deposition is also present in primary and metastatic brain tumors, CREKA-micelles were applied as targeting agents for gliomas [18–20]. The CMC of micelles was close to 1 μM, which is comparable to micelle systems composed of DSPE-PEG(2000) and 100 μl of 1 mM micelle solution was chosen to be administered in mice because the concentration remains above the CMC even upon injecting into approximately 2 mL of circulating blood volume (Fig. 1A) [21,25]. The conjugation of CREKA to DSPE-PEG(2000)-maleimide slightly increased the micelle size from 7.6 ± 1.2 to 8.2 ± 1.2 nm (Table 1, Fig. 1B). As particles from 5 to 250 nm are reported to exhibit higher transport efficiency to tumors, the nanoparticles obtained here are within the range of this requirement [8,26–28]. The addition of the peptide also changed the zeta potential of micelles from −27.4 ± 4.5 to −12.6 ± 1.6 mV. The surface charge of Cy7-micelles can be attributed to the phosphate group of the DSPE tail, PEG molecules, and the maleimide, which is increased by the addition of the arginine and lysine-containing peptide.

Cy7 was chosen as the fluorophore to be incorporated into micelles because its excitation and emission wavelength in the near infrared range is optimal to achieve both deep tissue penetration and low auto-fluorescence for *in vivo* imaging [29]. Fig. 4 confirmed Cy7-CREKA-micelles accumulate in the brain by 1 h and that the amount of accumulation can be monitored and quantified through *in vivo* imaging, supporting the possibility for quantitative diagnostics using this system. When brains were excised after 3 h, 24 h, and 7 days post-injection, fluorescence was detectable in tumors for both Cy7-micelles and Cy7-CREKA-micelles, confirming that the micelles passively target gliomas by the EPR effect and that the CREKA-modified micelles can further increase retention by active targeting (Fig. 5). Accumulation for both Cy7-micelles and Cy7-CREKA-micelles between 3 h and 24 h post-injection was not statistically significant although slightly higher at the latter time point, suggesting that our micelle delivery system has the potential to deliver the maximum amount of payload within 3 h after administration and is maintained for at least 24 h. Moreover, it is possible that all of the fibrin-binding sites of the tumor are occupied by 3 h; since micelles were constructed with a molar ratio of 10:90 for Cy7- and CREKA-containing amphiphiles, future drug delivery studies will alter the peptide amphiphile ratio to exploit micelle binding and offer the most efficient and highest amount of therapy to the tumor site.

After 7 days, micelles were cleared from the brain. Notably, no fluorescence signal was found when either Cy7-micelles or Cy7-CREKA-micelles were administered in a brain injury model. This confirms that the intracranial injection technique did not contribute to micelle accumulation to the brain, but instead, micelles target specifically to tumors via penetration of the compromised BBB. Upon histological examination, the fluorescence signal followed the optical imaging trends in which Cy7-CREKA-micelles accumulate in the tumor to a greater extent than Cy7-micelles (Fig. 6). Concentrated, fluorescent dots may represent micelles entrapped within the tumor blood vessels or thrombi, whereas dimmer regions of fluorescence may represent extravascular diffusion across the blood-tumor-barrier (BTB) of Cy7-CREKA-micelles into the glioma tissue due to increased accumulation (Fig. 1) [27]. In order to verify whether Cy7-CREKA-micelles bind to intravascular fibrin within gliomas, the presence of fibrin was determined via immunohistochemistry. However, upon processing tissue sections for fibrin detection, the Cy7 signal was quenched.

In addition to the influence of material properties of nanoparticles on the penetration and accumulation in tumor tissues, previous studies have reported particle size to affect the clearance mechanisms of nanoparticles [26,30–33]. Since both Cy7- and Cy7-CREKA-micelles are approximately 8 nm in diameter, they fall in the range of particles that are capable of both renal clearance via the kidneys and bladder and elimination by macrophages in the spleen and liver through the reticuloendothelial system (RES), which is consistent with the biodistribution data found in Fig. 7 [34,35]. By 7 days, the majority of the micelles were cleared and only remained within the liver confirming RES to play a larger role in clearance. H & E images confirmed that the liver and kidney, key organs involved in renal clearance and RES, showed no signs of tissue damage and had morphologies that were similar to that of PBS-treated controls, establishing the biocompatibility and safety of these micelles as a targeted drug delivery system for the treatment of glioblastoma.

## 5. Conclusions

Cy7-labeled micelles were modified with the fibrin-binding peptide, CREKA, to enhance the targeting of micelles to glioblastoma. Cy7-micelles and Cy7-CREKA-micelles were spherical in shape and the addition of the pentapeptide increased the average particle size and zeta potential from 7.6 to 8.2 nm, and −27.4 to −12.6 mV, respectively. Upon intravenous injection into a mouse model of intracranial glioma, Cy7-micelles passively accumulated at the tumor site via the EPR effect, whereas Cy7-CREKA-micelles displayed enhanced tumor homing via active targeting, confirmed by *in vivo* and *ex vivo* imaging and immunohistochemistry. Importantly, neither Cy7-micelles nor Cy7-CREKA-micelles localized to the brain in an injury model, conferring micelle specificity to brain tumors via penetration across the compromised BBB. Bio-distribution of micelles showed high accumulation in the liver and kidneys, which suggests both renal clearance and RES plays a role in micelle elimination and organs showed no signs of cytotoxicity or tissue damage determined via histological evaluation. Our study demonstrates a new avenue for targeting glioblastoma, and future studies will harness these capabilities to achieve highly efficient, chemotherapeutic drug delivery for the treatment of malignant gliomas.

## Figures and Tables

**Table 1:** Size and zeta potential of micelles.

	Cy7	Cy7-CREKA
Diameter (nm)	7.6 ± 1.2	8.2 ± 1.2
ZP (mV)	− 27.4 ± 4.5	− 12.6 ± 1.6
